# ATP Releasing Connexin 30 Hemichannels Mediate Flow-Induced Calcium Signaling in the Collecting Duct

**DOI:** 10.3389/fphys.2013.00292

**Published:** 2013-10-16

**Authors:** Per Svenningsen, James L. Burford, János Peti-Peterdi

**Affiliations:** ^1^Departments of Physiology and Biophysics, and Medicine, Zilkha Neurogenetic Institute, University of Southern CaliforniaLos Angeles, CA, USA; ^2^Department of Cardiovascular and Renal Research, Institute of Molecular Medicine, University of Southern DenmarkOdense, Denmark

**Keywords:** connexin, hemichannel, ATP release, calcium imaging, intravital microscopy

## Abstract

ATP in the renal tubular fluid is an important regulator of salt and water reabsorption via purinergic calcium signaling that involves the P2Y_2_ receptor, ENaC, and AQP2. Recently, we have shown that connexin (Cx) 30 hemichannels are localized to the non-junctional apical membrane of cells in the distal nephron-collecting duct (CD) and release ATP into the tubular fluid upon mechanical stimuli, leading to reduced salt and water reabsorption. Cx30^−/−^ mice show salt-dependent elevations in BP and impaired pressure-natriuresis. Thus, we hypothesized that increased tubular flow rate leads to Cx30-dependent purinergic intracellular calcium ([Ca^2+^]_i_) signaling in the CD. Cortical CDs (CCDs) from wild type and Cx30^−/−^ mice were freshly dissected and microperfused *in vitro*. Using confocal fluorescence imaging and the calcium-sensitive fluorophore pair Fluo-4 and Fura Red, we found that increasing tubular flow rate from 2 to 20 nl/min caused a significant 2.1-fold elevation in [Ca^2+^]_i_ in wild type CCDs. This response was blunted in Cx30^−/−^ CCDs ([Ca^2+^]_i_ increased only 1.2-fold, *p* < 0.0001 vs. WT, *n* = 6 each). To further test our hypothesis we performed CD [Ca^2+^]_i_ imaging in intact mouse kidneys *in vivo* using multiphoton microscopy and micropuncture delivery of the calcium-sensitive fluorophore Rhod-2. We found intrinsic, spontaneous [Ca^2+^]_i_ oscillations in free-flowing CDs of wild type but not Cx30^−/−^ mice. The [Ca^2+^]_i_ oscillations were sensitive also to P2-receptor inhibition by suramin. Taken together, these data confirm that mechanosensitive Cx30 hemichannels mediate tubular ATP release and purinergic calcium signaling in the CD which mechanism plays an important role in the regulation of CD salt and water reabsorption.

## Introduction

In the kidney, ATP released from the tubular epithelium regulates salt and water transport through activation of purinergic signaling along the nephron and collecting duct (CD). Renal epithelial [Ca^2+^]_i_ signaling and ATP release may be elicited by mechanical stimulation e.g., increased tubular fluid flow rate (Satlin et al., 2006; Jensen et al., 2007; Praetorius and Leipziger, 2009; Xu et al., 2009) which shows well-characterized physiological oscillations *in vivo* due to renal hemodynamic feedback mechanisms (Holstein-Rathlou and Leyssac, 1986; Marsh et al., 2005; Kang et al., 2006; Peti-Peterdi et al., 2009). Accordingly, the activation of flow-induced purinergic calcium signaling in renal and tubular epithelial cells including cells of the CCD has been described in detail (Woda et al., 2002; Jensen et al., 2007; Praetorius and Leipziger, 2009; Sipos et al., 2009; Xu et al., 2009). In terms of the luminal ATP release mechanism in the CD, the expression of connexin 30 (Cx30) hemichannels has been shown to be crucial and to play an integral role in regulating CD salt and water transport (Sipos et al., 2009; Stockand et al., 2010; Mironova et al., 2011).

Cx30 is a member of the connexin (Cx) family comprised by 21 structurally similar isoforms (Spray et al., 2006). The Cx family are transmembrane proteins that can form non-selective pores in the plasma membrane, allowing passage of molecules up to 1 kDa in mass (Spray et al., 2006). The classical view is that these pores align and form gap junctions in the junctional membrane, allowing the transfer of molecules between adjacent cells thereby facilitating intercellular communication. However, increasing evidence suggests that Cx pores also reside in non-junctional plasma membrane domains, where they form large, mechanosensitive ion channels which allow the passage of a variety of small molecules and metabolites including ATP (Cotrina et al., 1998; Ebihara, 2003; Bao et al., 2004). Cx isoforms have been identified in nearly all tissues (Willecke et al., 2002). They appear to be involved in a wide variety of physiological functions depending on the isoform expressed. In the kidney, the isoforms Cx37, Cx40 and Cx43 are localized in the vasculature, glomerulus, and tubular segments in a punctuated pattern, typical of classic gap junction channels (Barajas et al., 1994; Arensbak et al., 2001). Interestingly, Cx30 is expressed at the luminal membrane in a subset of cells in the distal nephron-CD in rat, rabbit, and mouse kidney (McCulloch et al., 2005; Sipos et al., 2009).

We recently reported that Cx30 knockout mice showed reduced luminal ATP release both *in vitro* (Sipos et al., 2009) and *in vivo* (Stockand et al., 2010; Mironova et al., 2011) leading to higher CD sodium reabsorption through the epithelial sodium channel (ENaC). Mice lacking the P2Y_2_ receptor also have increased renal tubular sodium reabsorption (Rieg et al., 2007). These findings suggest that auto-/paracrine effects of luminal ATP released via Cx30 hemichannels involve regulation of renal salt and water reabsorption and this novel mechanism appears to be integral in several physiological mechanisms including pressure natriuresis and diuresis which maintain body fluid balance and blood pressure (Sipos et al., 2009), and also aldosterone escape (Stockand et al., 2010) via the regulation of ENaC activity (Mironova et al., 2011).

We hypothesized that the high tubular flow-induced [Ca^2+^]_i_ signaling which is an established hallmark of the effects of local ATP release in the CD is Cx30-dependent. To test the hypothesis, we used fluorescence imaging techniques to visualize calcium signaling in isolated CCDs *in vitro* as well as in CDs of the living and intact kidney *in vivo* in wild-type or Cx30 knockout mice.

## Methods

### Mice

The Cx30^−/−^ mouse model was established and described previously (Teubner et al., 2003; Sipos et al., 2009). Wild-type and Cx30^−/−^ mice (C57BL6 background) were bred at the University of Southern California. All animal protocols were conducted in conformity with the Guiding Principles for Research Involving Animals and Human Beings and were approved by the Institutional Animal Care and Use Committee of the University of Southern California. Genotype was confirmed by PCR of tail biopsies.

### Collecting duct calcium imaging *in vitro*

CCDs were freshly dissected from mouse kidneys and loaded with the calcium-sensitive fluorophore pair Fluo-4 and Fura Red as previously described (Sipos et al., 2009). The intact tubules were cannulated and microperfused using a set of concentric glass micropipettes in the 2 to 20 nl/min range with a solution containing (in mM) 25 NaCl, 5 KCl, 1 MgSO_4_, 1.6 NaHPO_4_, 0.4 NaH_2_PO_4_, 5 d-glucose, 1.5 CaCl_2_, 110 NMDG-cyclamate, and 10 HEPES as described and shown previously for renal arteriolar and tubular segments (Peti-Peterdi, 2006; Sipos et al., 2009, 2010). The calibration of perfusion pressure and the resulting tubular flow rate was reported earlier (Peti-Peterdi, 2006). Fluo-4 (excitation at 488 nm, emission at 520 ± 20 nm) and Fura Red (excitation at 488 nm, emission at >600 nm) fluorescence was detected using a Leica TCS SP2 AOBS MP confocal microscope system, and fluorescence was calibrated to [Ca^2+^]_i_ as described previously (Peti-Peterdi, 2006).

### Micropuncture

Mice were anesthetized using ketamine and xylazine ip (100 and 10 mg/kg, respectively) and were surgically prepared for renal micropuncture and tubular microperfusion delivery of the calcium-sensitive fluorophore Rhod-2AM (10 μM, Invitrogen). The mice were placed on a homoeothermic operating table (Vestavia Scientific) and whole body temperature was kept at ~37°C. A ~22 mm dorsal incision on the left subcostal flank and two horizontal midline incisions were made for placement of kidney cup. The left kidney was gently exteriorized and renal pedicle and kidney cleanly dissected of debris and fat. A 22 mm kidney cup (Vestavia Scientific) was placed within the retracted peritoneal cavity opening and the left kidney was placed within the kidney cup and continuously bathed with warm 0.9% saline. Glass capillary tubes (“ID 0.084 × 0.064,” Drummond Scientific Company) were pulled with a PP-830 pipette puller (Narishige), tip grinded to an O.D. of ~2 μm, filled with the fluorophore Rhod-2 and put on a micromanipulator (Leitz). Tubule segments were selected using a Stemi 200 stereomicroscope (Zeiss, 250 × magnification), micropunctured with the glass micropipette and microperfused with the fluorescent dye at a rate of 5–10 nl/min for 15–20 min.

### Collecting duct calcium imaging *in vivo*

The mice were placed on the stage of a Leica TCS SP5 AOBS MP confocal microscope system powered by a Chameleon Ultra-II MP laser (Coherent Inc.) and a DMI 6000 inverted microscope and the exposed kidney was placed in a coverslip-bottomed heated chamber bathed in normal saline. The kidney was visualized from below as described before (Kang et al., 2006; Sipos et al., 2007). Rhod-2 was excited at 860 nm and the emitted Rhod-2 fluorescence was collected using a TRITC filter and external detectors. During all procedures, core body temperature was maintained by using a homeothermic table. In some experiments the apparent xy-movements of the kidney during time-lapse imaging were corrected using the TurboReg plug-in for ImageJ (Thévenaz et al., 1998). Analysis of the xyt time-lapse image sequences was performed *post-hoc* by placing ROIs over those CD cells which were intensely labeled by Rhod–2, usually in the cell nucleus. Suramin (10 mg/kg, Sigma) was infused via the cannulated carotid artery at 10 μl/min.

### Statistical analysis

Data are shown as average ± s.e.m. Students *t*-test was used for statistical comparison of two groups, and 1-Way ANOVA was performed with *post-hoc* Bonferroni multiple comparison test for the comparison of 3 groups. *P* < 0.05 was considered significant.

## Results

### Cx30 dependence of the flow-induced [Ca^2^+]_i_ response in the isolated *in vitro* microperfused CCD

**Figure 1:**
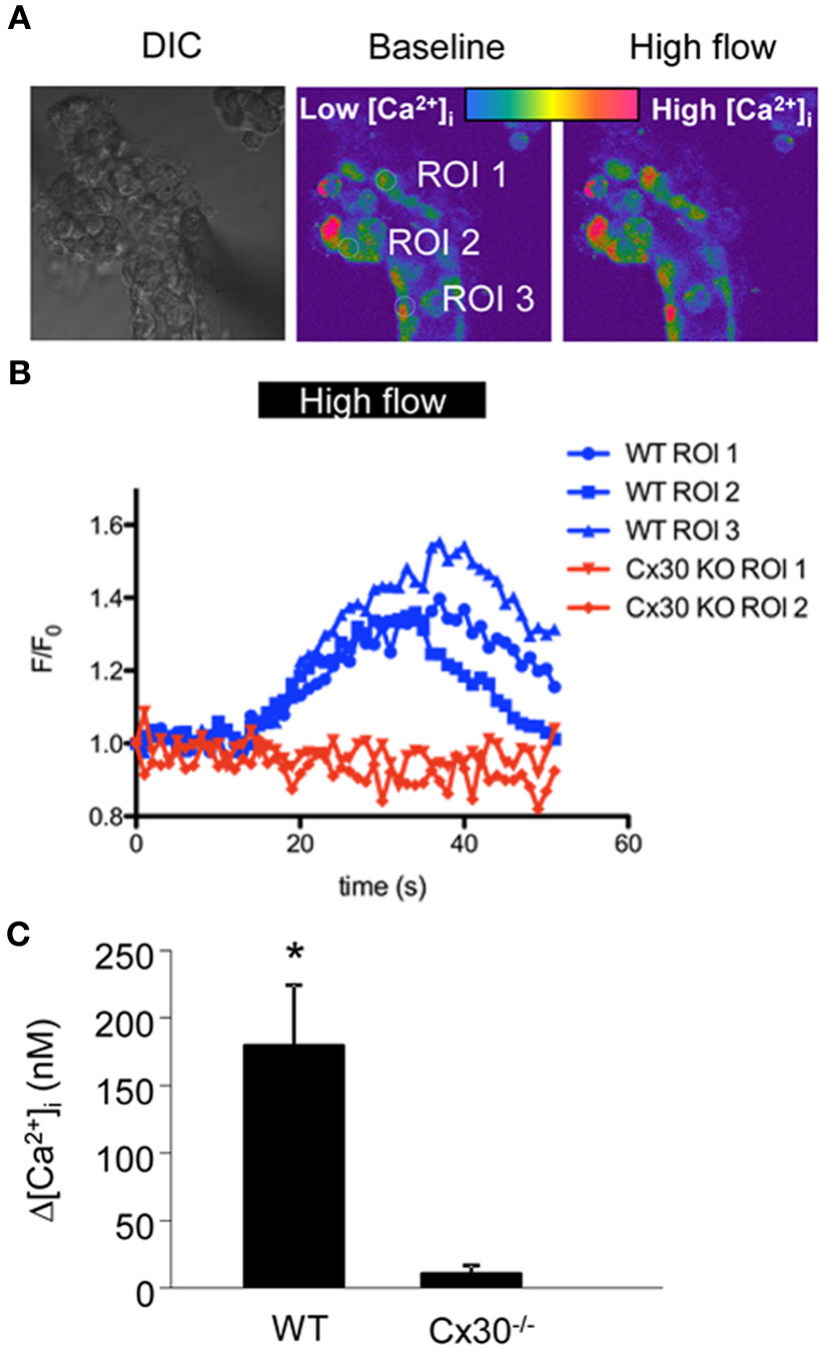
**Flow-induced increases in [Ca^2+^]_i_ in cells of the *in vitro* microperfused collecting duct. (A)** CCDs from wild type and Cx30^−/−^ mice were freshly dissected and microperfused *in vitro*, and loaded with the Ca^2+^-sensitive fluorophore pair Fluo-4 and Fura Red. Left: DIC image of a representative preparation. Middle, and right: Pseudo-colored confocal Fluo-4/Fura Red ratio images of the same CCD before and after increasing tubular flow from low (2 nl/min) to high (20 nl/min) rates. **(B)** Representative recordings of Fluo-4/Fura Red ratio from 3 ROIs from wild type (blue, as shown in panel **A**) and 2 ROIs from Cx30^−/−^ CCDs (red) show that Cx30 is essential for flow-induced increases in [Ca^2+^]_i_. **(C)** Summary of the CCD [Ca^2+^]_i_ responses illustrates that the increase in [Ca^2+^]_i_ in response to high flow was almost completely abolished in Cx30^−/−^ mice. (^*^ *p* < 0.001, *n* = 10 wild-type and 8 Cx30^−/−^).

To test our hypothesis that increased flow rates in the CD lead to Cx30-mediated increases in [Ca^2+^]_i_, we freshly dissected and microperfused CCDs *in vitro* from wild-type and Cx30^−/−^ mice and loaded them with calcium fluorophores (Figure 1A). Under conditions of baseline low flow (2 nl/min), fluorescence [Ca^2+^]_i_ imaging did not detect significant differences in [Ca^2+^]_i_ in CCD cells between wild-type and Cx30^−/−^ mice (286 ± 34 nM and 279 ± 44 nM, respectively, *n* = 10 wild-type and 8 Cx30^−/−^). In response to increasing flow rate to 20 nl/min, CCDs from wild-type mice produced a significant increase in [Ca^2+^]_i_ (average Δ[Ca^2+^]_i_ was 177 ± 45 nM, *p* < 0.05, *n* = 10), whereas no significant changes were detected in CCDs from Cx30^−/−^ mice (average Δ[Ca^2+^]_i_ was 9 ± 6 nM, *n* = 8) (Figures 1A–C). Thus, our data suggest that the flow-induced increases in [Ca^2+^]_i_ *in vitro* microperfused CCDs were dependent on the expression of Cx30.

### CD [Ca^2^+]_i_ imaging *in vivo*

**Figure 2:**
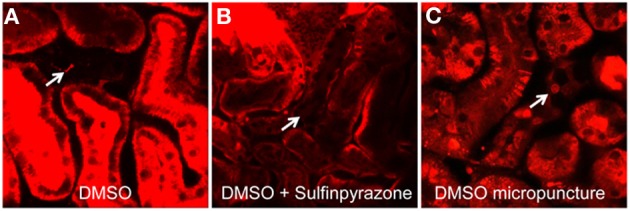
**Micropuncture delivery of Rhod-2 results in effective loading of the CD *in vivo*.** Loading of the cells of the CD (arrows) with the calcium fluorophore Rhod-2 *in vivo* was established by first injecting Rhod-2 dissolved in DMSO alone **(A)**, or together with Sulfinpyrazone **(B)** under the renal capsule. Neither strategies resulted in cellular dye uptake in the CD. However, loading of CDs (arrow) were achieved by using micropuncture delivery of Rhod-2 dissolved in DMSO **(C)**.

In order to test if Cx30 is involved in CD [Ca^2+^]_i_ signaling *in vivo*, we first developed a technique to efficiently load CD cells with the commercially available cell permeable acetoxymethylester (AM) form of fluorophores for confocal fluorescence imaging. The calcium-sensitive fluorophore Rhod-2 was employed in the present experiments, as red fluorescent probes are preferred over green dyes due to the low overlap of their emission with the significant renal tissue autofluorescence. Initially, we used the multicell bolus loading (MCBL) technique that we previously described for real-time imaging of intracellular pH and [Ca^2+^]_i_ in the proximal tubules (Sipos et al., 2007; Peti-Peterdi et al., 2009). Injection of Rhod-2 dissolved in DMSO directly under the renal capsule resulted in very poor labeling of the cells of the CD (Figure 2A, arrow). To test if the poor loading of the CDs were due to dye leakage via organic anion transporters, we supplemented the Rhod-2/DMSO solution with the organic anion transporter inhibitor sulfinpyrazone to reduce leakage of the de-esterified form of Rhod-2. However, as shown in Figure 2B sulfinpyrazone supplementation did not increase the CD loading of Rhod-2. To achieve a more efficient labeling, we next tried tubular/interstitial micropuncture delivery of Rhod-2. As shown in Figure 2C, this loading strategy resulted in effective Rhod-2 loading of the cells in the CD, and this loading strategy was employed in the subsequent experiments.

### Cx30 dependence of CD [Ca^2^+]_i_ signaling *in vivo*

**Figure 3:**
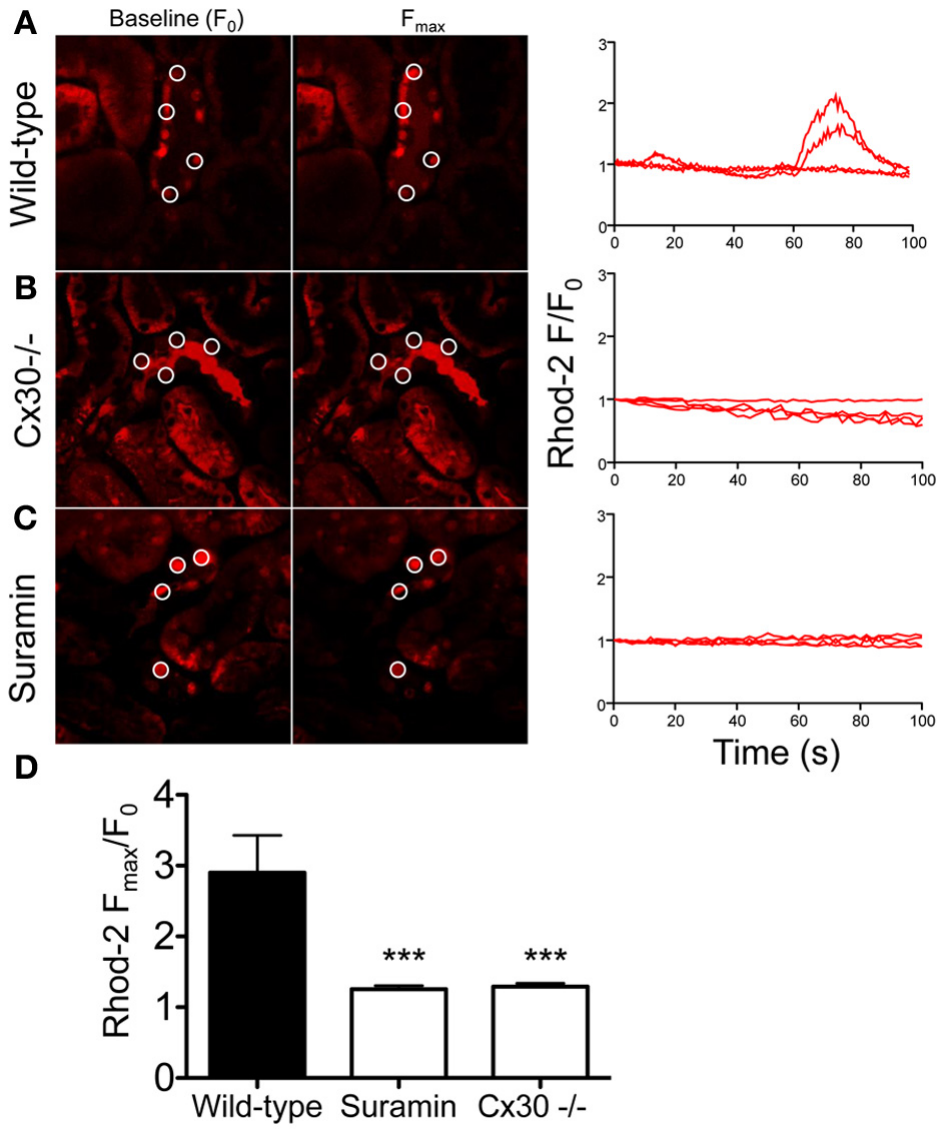
**Multiphoton imaging of CD cell [Ca^2+^]_i_ signaling in the intact mouse kidney *in vivo*.** Cells of the CD were loaded with the fluorescent calcium indicator Rhod-2 using micropuncture delivery and its fluorescence intensity (*F*) was recorded within intracellular regions of interest over time, without exogenous stimuli. The CD was identified based on anatomical (heterogenous cell population with typical, bulging luminal surface, downstream from the Y-shaped junction of adjacent connecting segments) and functional (highly concentrated tubular fluid based on its high fluorescence intensity) considerations. Representative images of the same preparation at baseline (*F*_0_) and at peak *F* values are shown and selected ROIs of CD cells are indicated by circles. Recordings of normalized Rhod-2 fluorescence (*F*/F_0_) demonstrate the presence of spontaneous [Ca^2+^]_i_ oscillations in a subset of cells of the CD in wild-type mice **(A)**. In contrast, low and steady-state Rhod-2 fluorescence was found in CD cells from Cx30^−/−^ mice **(B)** or in wild-type mice after suramin treatment **(C)**. Summary of the CD [Ca^2+^]_i_ responses **(D)** illustrates that the spontaneous [Ca^2+^]_i_ oscillations were almost completely abolished in Cx30^−/−^ mice or after suramin treatment. (^***^p < 0.001 vs. wild-type, *n* = 5 wild-type, 7 suramin, and 11 Cx30^−/−^).

To further support our hypothesis that flow-induced [Ca^2+^]_i_ signaling in the CD is dependent on Cx30 expression, we used *in vivo* multiphoton microscopy of calcium fluorophore-loaded intact kidneys in wild-type and Cx30^−/−^ mice. Spontaneous [Ca^2+^]_i_ oscillations in the free-flowing CDs were measured which were likely the result of intrinsic tubular flow oscillations caused by tubuloglomerular feedback and the myogenic mechanism as described before (Holstein-Rathlou and Leyssac, 1986; Marsh et al., 2005; Peti-Peterdi et al., 2009). Although tubular flow was not measured or correlated with [Ca^2+^]_i_ in the present studies, temporary increases in CD flow were evident by the alterations in CD tubular diameter (not shown). In kidneys from wild-type mice a subset of CD cells showed spontaneous oscillations in Rhod-2 fluorescence reflecting changes in [Ca^2+^]_i_ (Figure 3A). In contrast, CD cells in Cx30^−/−^ mice showed steady-state Rhod-2 fluorescence with no signs of oscillations (Figures 3B,D). To test if the observed CD [Ca^2+^]_i_ oscillations in wild-type mice were due to P2-receptor signaling, the purinergic receptor inhibitor suramin was administered in wild-type mice. As shown in Figures 3C,D, suramin treatment abolished the oscillations in Rhod-2 fluorescence and resulted in steady-state CD [Ca^2+^]_i_ levels (average Δ*F*_max_/*F*_0_ was 2.90 ± 0.53, 1.26 ± 0.05, and 1.29 ± 0.04 in wild-type, suramin-treated, and Cx30^−/−^ mice, respectively, *p* < 0.001 Cx30^−/−^ and suramin groups vs. wild-type, *n* = 5 wild-type, 7 suramin, and 11 Cx30^−/−^).

## Discussion

This study is a direct and logical continuation of our recent line of research which aims to characterize ATP releasing mechanisms and their physiological relevance in the kidney. In the present experiments we used confocal fluorescence imaging approaches to further examine the function of one particular ATP conduit, the Cx30 hemichannel in the renal CD. Using *in vitro* microperfused CCDs and ratiometric calcium imaging we first found that the classic phenomenon of tubular flow-induced increases in CD [Ca^2+^]_i_ was dependent on the expression of Cx30. In addition, spontaneous [Ca^2+^]_i_ oscillations in free-flowing CDs were observed in the intact living kidney of wild type but not Cx30^−/−^ mice and the [Ca^2+^]_i_ responses were found to be sensitive to suramin, a P2 purinergic receptor inhibitor. Our results are consistent with the presence of mechanosensitive, ATP releasing Cx30 hemichannels in the distal nephron-CD system and established that CD cell [Ca^2+^]_i_ responses are an integral element of the intra-tubular autocrine/paracrine purinergic system in the CD that is driven by Cx30-mediated ATP release. The present studies also established the exciting new technical advance of directly and quantitatively visualizing CD [Ca^2+^]_i_ responses in the intact mouse kidney *in vivo*.

The present findings are in line with our previous study showing that Cx30-mediated ATP release in the CD can be triggered by mechanical forces, for example by increases in tubular fluid flow rate (Sipos et al., 2009). In contrast to this previous work in which exogenous biosensor cell [Ca^2+^]_i_ responses were measured as an indicator of ATP release (Sipos et al., 2009), the present studies measured Cx30-dependent [Ca^2+^]_i_ responses in cells of the intact microperfused CCD (Figure 1). Flow-induced [Ca^2+^]_i_ responses and ATP release in renal and tubular epithelial cells including the CD are well established (Woda et al., 2002; Liu et al., 2003; Satlin et al., 2006; Jensen et al., 2007; Praetorius and Leipziger, 2009; Sipos et al., 2009; Xu et al., 2009) and the downstream effects of ATP involve purinergic signaling through binding to P2X and P2Y purinergic receptors (Schwiebert and Kishore, 2001; Unwin et al., 2003) that regulate salt and water reabsorption. Consistent with Cx30-mediated mechanosensitive ATP release, recent studies found reduced pressure natriuresis and diuresis in Cx30^−/−^ mice (Sipos et al., 2009). Cx30^−/−^ mice also show reduced ATP release into the tubular fluid and urine and display ENaC hyperactivity in response to a high sodium intake (Sipos et al., 2009; Mironova et al., 2011). It should be noted that Cx30^−/−^ mice appear to express an unaltered level of P2 receptor transcripts (Sipos et al., 2009) as well as functional P2 receptors in the collecting ducts, confirmed by the potent inhibitory effect of exogenous ATP on ENaC activity (Mironova et al., 2011). Collectively, these findings indicate the Cx30^−/−^ mice display a disrupted paracrine feedback inhibition of ENaC by ATP due to a failure in ATP release rather than in downstream ATP signaling. Elevated [Ca^2+^]_i_ is one of the many known signaling pathways of purinergic ATP receptors including the activation of the P2Y_2_ receptor (Vallon and Rieg, 2011) which inhibits CD sodium and water reabsorption (Pochynyuk et al., 2008). The present studies further confirm the functional expression and *in vivo* relevance of ATP releasing Cx30 hemichannels in the CD and suggest the involvement of Cx30-dependent autocrine/paracrine [Ca^2+^]_i_ responses in the function of the intra-tubular purinergic system (i.e., inhibition of salt and water transport).

**Figure 4:**
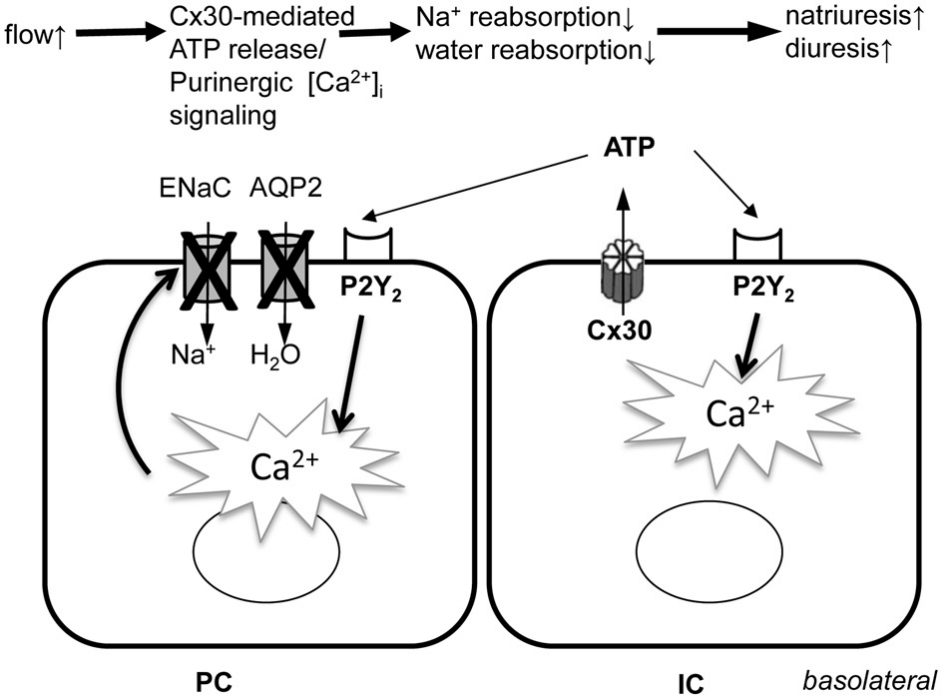
**Illustration of the role of Cx30-mediated ATP release and subsequent purinergic [Ca^2+^]_i_ signaling in the function of the intra-tubular autocrine/paracrine purinergic system in the CD.** PC, principal cell; IC, intercalated cell; ENaC, epithelial Na^+^ channel; AQP2, aquaporin 2 water channel; Cx30, connexin 30 hemichannel; P2Y_2_, ATP receptor subtype.

The present study revealed the highly dynamic nature of [Ca^2+^]_i_ changes in cells of the CD *in vivo* (Figure 3) suggesting rapid, temporary oscillations in the regulation and rate of salt and water reabsorption in cells of the CD. Since no exogenous stimuli were applied in these experiments, the Cx30-dependent spontaneous oscillations in CD [Ca^2+^]_i_ were likely the result of the endogenous tubular fluid flow oscillations (triggering mechanosensitive Cx30-mediated ATP release) that are known to exist due to renal hemodynamic feedback mechanisms (Holstein-Rathlou and Leyssac, 1986; Marsh et al., 2005; Kang et al., 2006; Peti-Peterdi et al., 2009). In our previous work we described and visually demonstrated (see supplementary videos 2 and 5) the presence of these endogenous tubular flow oscillations, and the simultaneous, phase-matched oscillations in proximal tubule diameter and [Ca^2+^]_i_ responses (Peti-Peterdi et al., 2009). In the present work, similar, simultaneous increases in [Ca^2+^]_i_ were detected *in vivo* in the CD (Figure 3A) and these responses were diminished in Cx30^−/−^ and suramin-treated mice (Figures 3B–D). The *in vitro* microperfusion experiments in which equal rates of high flow were applied in both wild-type and Cx30^−/−^ CDs (Figure 1) suggested that the diminished CD [Ca^2+^]_i_ responses *in vivo* were not due to the lack of flow stimulation but rather the failure in ATP release in Cx30^−/−^ mice. Therefore, the physiological oscillations in tubular fluid flow may function as an important endogenous diuretic mechanism that involves Cx30-mediated ATP release, purinergic [Ca^2+^]_i_ signaling and inhibition of tubular salt and water reabsorption in the CD. Consistent with this, Cx30^−/−^ mice which lack this mechanism feature a salt retention phenotype and have salt-sensitive hypertension (Sipos et al., 2009; Mironova et al., 2011). P2Y_2_ receptor knockout mice display a similar phenotype (Rieg et al., 2007), further supporting the view that dynamic purinergic mechanisms in the CD play an important role in the (patho)physiological regulation of salt and water balance. Figure 4 illustrates the elements of this local purinergic sensory, signaling and regulatory system in the CD including Cx30-dependent ATP release and [Ca^2+^]_i_ signaling that the present studies established.

It should be noted that the present studies detected *in vivo* [Ca^2+^]_i_ responses only in a subset of CD cells rather than globally. However, this was likely the result of technical limitations (unequal loading of cells and cortical tissue regions with Rhod-2) since in the *in vitro* dissected and microperfused CD, in which fluorophore loading was very efficient, all cells produced flow-induced [Ca^2+^]_i_ responses (Figure 1). Also, in the present *in vivo* experiments we did not perform long-term time-lapse [Ca^2+^]_i_ imaging due to the above technical limitations, therefore, we did not address the more complex features of CD [Ca^2+^]_i_ signals (such as frequency and regularity of oscillations, cell-to-cell propagation, synchronization, etc. and their importance in CD function). However, these important questions need to be addressed in future work using more suitable experimental approaches (e.g., new mouse models with renal tubular expression of genetically encoded calcium indicators).

These studies were carried out in mice, where the expression of Cx30 is restricted to the intercalated cells of the CNT and CCD (McCulloch et al., 2005). However, in kidneys from rat and rabbits Cx30 was also expressed in other nephron segments including the thick ascending limb (McCulloch et al., 2005), indicating that Cx30-mediated ATP release and purinergic [Ca^2+^]_i_ signaling could be regulating epithelial transport processes in other tubular segments in other species. In addition, the ATP channel pannexin 1 which was recently localized to the apical plasma membrane of several renal tubule segments including the CD (Hanner et al., 2012) may play a similar role. In terms of other flow-sensing mechanisms in renal tubular cells, in our recent report we speculated (Sipos et al., 2009) that apical membrane Cx30 hemichannel opening induced by mechanical stimulation (interstitial pressure, tubular flow) may involve the supportive function of primary cilia (in principal cells) and microvilli (in intercalated cells) that are well-established sensors of shear and hydrodynamic impulses (Liu et al., 2003). Supporting the existence of interaction between the intra-tubular ATP purinergic system and primary cilia function are the recent findings that the loss of apical monocilia on renal tubular epithelial cells impairs ATP secretion across the apical cell surface (Hovater et al., 2008; Praetorius and Leipziger, 2009; Xu et al., 2009).

In summary, the present studies successfully visualized [Ca^2+^]_i_ responses in the CD in the intact kidney *in vivo* and further characterize the function of ATP releasing Cx30 hemichannels in the mouse CD. Our results established that Cx30-dependent [Ca^2+^]_i_ responses are an integral element of the intra-tubular autocrine/paracrine purinergic system in the CD that plays an important role in the regulation of renal salt and water reabsorption, the maintenance of body fluid and electrolyte balance and normal blood pressure.

## Conflict of interest statement

The authors declare that the research was conducted in the absence of any commercial or financial relationships that could be construed as a potential conflict of interest.
